# Divalent Metal Ion-Induced Folding Mechanism of RNase H1 from Extreme Halophilic Archaeon *Halobacterium* sp. NRC-1

**DOI:** 10.1371/journal.pone.0109016

**Published:** 2014-09-30

**Authors:** Elias Tannous, Shigenori Kanaya

**Affiliations:** Department of Material and Life Science, Graduate School of Engineering, Osaka University, Suita, Osaka, Japan; University of North Carolina at Chapel Hill, United States of America

## Abstract

RNase H1 from *Halobacterium* sp. NRC-1 (Halo-RNase H1) is characterized by the abundance of acidic residues on the surface, including bi/quad-aspartate site residues. Halo-RNase H1 exists in partially folded (I) and native (N) states in low-salt and high-salt conditions respectively. Its folding is also induced by divalent metal ions. To understand this unique folding mechanism of Halo-RNase H1, the active site mutant (2A-RNase H1), the bi/quad-aspartate site mutant (6A-RNase H1), and the mutant at both sites (8A-RNase H1) were constructed. The far-UV CD spectra of these mutants suggest that 2A-RNase H1 mainly exists in the I state, 6A-RNase H1 exists both in the I and N states, and 8A-RNase H1 mainly exists in the N state in a low salt-condition. These results suggest that folding of Halo-RNase H1 is induced by binding of divalent metal ions to the bi/quad-aspartate site. To examine whether metal-induced folding is unique to Halo-RNase H1, RNase H2 from the same organism (Halo-RNase H2) was overproduced and purified. Halo-RNase H2 exists in the I and N states in low-salt and high-salt conditions respectively, as does Halo-RNase H1. However, this protein exists in the I state even in the presence of divalent metal ions. Halo-RNase H2 exhibits junction ribonuclease activity only in a high-salt condition. A tertiary model of this protein suggests that this protein does not have a quad-aspartate site. We propose that folding of Halo-RNase H1 is induced by binding of divalent metal ion to the quad-aspartate site in a low-salt condition.

## Introduction

RNase H is an enzyme that cleaves the RNA strand of RNA/DNA hybrid [1]. It cleaves the P-O3’ bond of the substrates with a two-metal-ion catalysis mechanism, in which two metal ions, such as Mg^2+^ and Mn^2+^, are directly involved in the catalytic function [2]. RNase H in its two types [3]–[5] is a key component for the growth and survival of all organisms [6]–[8]; while type 1 RNase H (RNase H1) plays a crucial role in DNA replication by removing the RNA primer of Okazaki fragments [8]–[10], type 2 RNase H (RNase H2) is believed to be more involved in DNA repair by removing the single ribonucleotides incorporated in the DNA [6], [11]–[17]. Both type 1 and type 2 RNases H are involved in RNA transcription by resolving the R-loops that block the proceeding of the replication fork, thus maintaining the integrity of the genome [18]–[21]. In addition, type 1 RNases H from retroviruses, which exist as a C-terminal domain of reverse transcriptases, are required for proliferation of retroviruses and therefore RNase H from human immunodeficiency virus type-1 (HIV-1) is regarded as a target for AIDS therapy [22].

Similar to various organisms studied to date, the extreme halophilic archaeon *Halobacterium* sp. NRC-1 possesses in its genome a single *rnhA* gene (Vng0255c) and a single *rnhB* gene (Vng1984G), encoding type 1 (Halo-RNase H1) and type 2 (Halo-RNase H2) RNases H respectively [23]. Halo-RNase H1, which is identified as the first archaeal type 1 enzyme, cleaves not only a typical RNA/DNA hybrid at the phosphodiester bonds of an RNA strand but also an Okazaki fragment-like substrate at the 3′-side of the ribonucleotide of the (5′)RNA-DNA(3′) junction [24]. The latter activity is different from the junction ribonuclease (JRNase) activity of type 2 RNases H (RNases H2), which catalyzes the cleavage of an Okazaki fragment-like substrate [25] and double-stranded DNA containing a single ribonucleotide [14], [15], [26], [27] at the 5′-side of the ribonucleotide of the (5′)RNA-DNA(3′) junction. In contrast, it remains to be determined whether Halo-RNase H2 exhibits activity.

Halo-RNase H1 consists of an N-terminal domain (residues 1–68) and a C-terminal RNase H domain (residues 69–199) [24]. The role of the N-terminal domain remains to be understood, because removal of this domain does not significantly affect the activity, stability, folding, and substrate binding affinity of Halo-RNase H1 [28]. Folding of Halo-RNase H1 has been studied using CD spectroscopy [28]. Halo-RNase H1 exists in partially folded (I) and native (N) states in low-salt and high-salt conditions respectively. However, it exists in the N state in the presence of ≥5 mM MnCl_2_ or ≥300 mM MgCl_2_ even in a low-salt condition. In the presence of the lower concentrations of these metal ions, Halo-RNase H1 exists in equilibrium between the I and N states. The fraction of the N state decreases as the concentration of these metal ions decreases. According to the crystal structure of Halo-RNase H1 folded in the presence of manganese ions (PDB code 4NYN), the protein surface is negatively charged due to the abundance of acidic residues. Three bi-aspartate sites are present on the surface, two of which are located close to each other to form a quad-aspartate site. These results suggest that negative charge repulsion on the surface that prevents folding of Halo-RNase H1 in a low-salt condition is suppressed by binding of divalent metal ions. However, the Halo-RNase H1 structure mentioned above does not clearly show the metal-binding site(s) that is/are responsible for folding of Halo-RNase H1 in a low-salt condition, because one protein molecule contacts three other molecules through manganese ions due to an artifact of the crystal packing. At any interface of these molecules, two manganese ions are coordinated with the active site residues (Asp75, Glu115, Asp139, and Asp189) of one molecule and one bi-aspartate site residue (Asp198) or two quad-aspartate site residues (Asp173 and Asp174) of the other. Therefore, it would be informative to examine whether the mutations that suppress negative charge repulsion at the active and bi/quad-aspartate sites affect folding of Halo-RNase H1 in a low salt-condition. It would also be informative to examine whether folding of Halo-RNase H2 is induced by divalent metal ions.

In this study, we showed that the mutations at the active site do not significantly affect the structure of Halo-RNase H1 in a low-salt condition; whereas, those at the bi/quad-aspartate sites considerably change it, in such a way that the fraction of the N state increases. In addition, we showed that, unlike Halo-RNase H1, Halo-RNase H2 exists in the I state even in the presence of divalent metal ions in a low-salt condition. It exists in the N state and exhibits JRNase activity only in a high-salt condition. A model of the Halo-RNase H2 structure shows that Halo-RNase H2 has two bi-aspartate sites on the surface, but does not have a quad-aspartate site. We propose that folding of Halo-RNase H1 is induced by binding of divalent metal ion to the quad-aspartate site in a low-salt condition.

## Results and Discussion

### Construction of Halo-RNase H1 mutants

**Figure 1:**
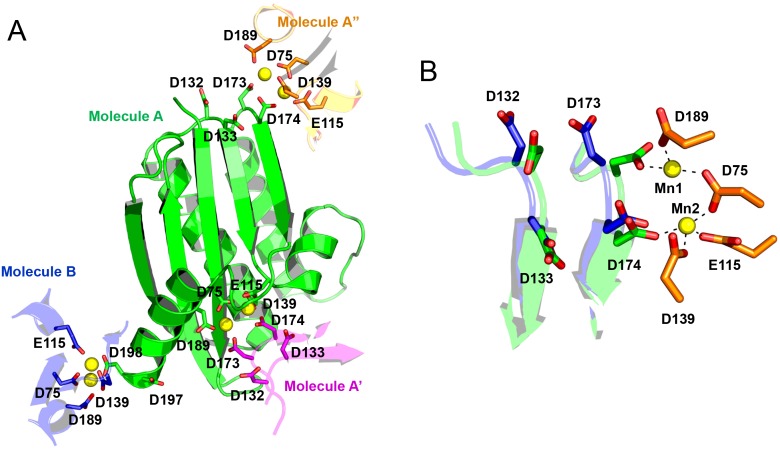
The crystal structure of Halo-RNase H1 (PDB code 4NYN). (A) The entire structure of molecule A (green) and a part of the structures of molecules B (blue), A′ (magenta) and A″ (orange) are shown. The side chains of the four acidic active site residues (Asp75, Glu115, Asp139, Asp189), bi-aspartate site residues (Asp197, Asp198), and quad-aspartate site residues (Asp132, Asp133, Asp173, Asp174) of molecule A are shown as green sticks, in which the oxygen atom is colored red. The side chains of the four acidic active site residues of molecules B and A″ are shown as blue and orange sticks respectively, in which the oxygen atom is colored red. The side chains of the quad-aspartate site residues of molecule A′ are shown as magenta sticks, in which the oxygen atom is colored red. Two manganese ions are shown as yellow spheres. (B) The structure of the quad-aspartate site of molecule A (green), which contacts the active site of molecule A″ (orange) through two manganese ions (Mn1 and Mn2), is superimposed onto that of molecule B (blue), which does not contact another molecule. The side chains of the four quad-aspartate site residues and four acidic active site residues, and two manganese ions are shown as in (Fig. 1A).

According to the crystal structure of Halo-RNase H1 folded in the presence of manganese ions (PDB code 4NYN), molecule A of Halo-RNase H1 contacts three other molecules (molecules B, A′, and A″) at the active, bi-aspartate, and quad-aspartate sites through two manganese ions (Fig. 1A). This is due to an artifact of the crystal packing, because size exclusion chromatography of Halo-RNase H1 in the presence of manganese ions suggests that the protein is monomeric in solution (data not shown). Molecules A and B contact each other within the crystal lattice. Molecules A and A′ or A″ contact each other at the interface between two different crystal lattices. At the interface between molecules A and B, two manganese ions are coordinated with the active site residues of molecule B and one bi-aspartate site residue (Asp198) of molecule A. At the interface between molecules A and A′ or A″, two manganese ions are coordinated with the active site residues of molecule A and two quad-aspartate site residues (Asp173 and Asp174) of molecule A′ or two quad-aspartate site residues of molecule A and the active site residues of molecule A″. This result suggests that folding of Halo-RNase H1 is induced by binding of manganese ions to the active, bi-aspartate, and/or quad-aspartate sites. Binding of manganese ions to those sites probably suppresses negative charge repulsion at those sites, which prevents folding of Halo-RNase H1 in a low-salt condition. To examine whether suppression of negative charge repulsion at all of these sites is necessary to induce folding of Halo-RNase H1, Halo-RNase H1 mutants, 2A-, 6A- and 8A-RNases H1, in which two of the four acidic active site residues (Asp75 and Asp139), six bi/quad-aspartate site residues (Asp132, Asp133, Asp173, Asp174, Asp197, and Asp198), and all of these aspartate residues are replaced by Ala, respectively, were constructed. These mutants were purified to give a single band on SDS-PAGE (data not shown).

### Far-UV CD spectra of Halo-RNase H1 mutants

**Figure 2:**
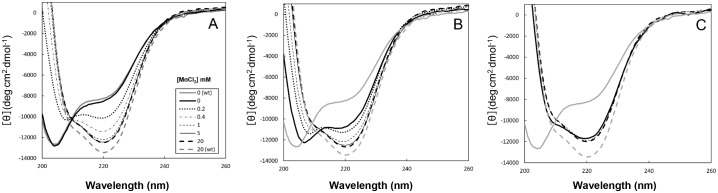
CD spectra of Halo-RNase H1 and its mutants. The far-UV CD spectra of 2A-RNase H1 (A), 6A-RNase H1 (B), and 8A-RNase H1 (C) measured at 25°C and pH 8.0 in the absence of salt and presence of various concentrations of MnCl_2_ are shown in comparison with those of Halo-RNase H1 measured in the absence of salt and divalent metal ions (*grey solid thick line*) and in the absence of salt and the presence of 20 mM MnCl_2_ (*grey dashed thick line*). MnCl_2_ concentrations: 0 mM (*solid thick line*); 0.2 mM (*dashed-dotted thin line*); 0.4 mM (*dotted thick line*); 1 mM (*dashed thin line*); 5 mM (*solid thin line*); 20 mM (*dashed thick line*).

Folding of 2A-, 6A- and 8A-RNases H1 was analyzed by CD spectroscopy. The far-UV CD spectra of 2A-RNase H1 (Fig. 2A), 6A-RNase H1 (Fig. 2B) and 8A-RNase H1 (Fig. 2C) measured in the presence of various concentrations of MnCl_2_ are compared with those of Halo-RNase H1 measured in the absence and presence of 20 mM MnCl_2_. All spectra were measured in the absence of salt. The spectra of Halo-RNase H1 measured in the absence and presence of 20 mM MnCl_2_ represent those of the Halo-RNase H1 proteins in the I and N states respectively [28]. In both conditions, Halo-RNase H1 exists in equilibrium between the I and N states. However, the fraction of the N state is 0% in the absence of MnCl_2_ and 100% in the presence of 20 mM MnCl_2_.

The spectrum of 2A-RNase H1 measured in the absence of MnCl_2_ is similar to that of Halo-RNase H1, whereas those of 6A- and 8A-RNases H1 are different, which suggest that the mutations at the active site of Halo-RNase H1 do not significantly affect the structure of Halo-RNase H1, while those at the bi/quad-aspartate sites significantly change it in such a way that the fraction of the N state increases in the absence of MnCl_2_. These spectra are changed in the presence of MnCl_2_, in such a way that the [θ] values at 205 and 220 nm increase and decrease respectively, as the MnCl_2_ concentration increases. The [θ] value of 2A-RNase H1 at 220 nm decreases from approximately −8000 to −12000 when the MnCl_2_ concentration increases to ≥1 mM, suggesting that the fraction of this protein in the N state is nearly 100% in the presence of ≥1 mM MnCl_2_. We have previously shown that higher concentrations (≥5 mM) of MnCl_2_ are required to increase the fraction of the Halo-RNase H1 protein in the N state to 100% [28]. This result suggests that suppression of negative charge repulsion at the active site increases the binding affinity of manganese ion(s) to the bi/quad-aspartate sites of Halo-RNase H1, because manganese ion(s) apparently binds/bind to the bi/quad-aspartate sites of 2A-RNase H1 more strongly than to those sites of Halo-RNase H1. Likewise, the [θ] value of 6A-RNase H1 at 220 nm decreases from approximately −11000 to −12500 when the MnCl_2_ concentration increases to ≥5 mM, suggesting that the fraction of this protein in the N state is nearly 100% in the presence of ≥5 mM MnCl_2_. The [θ] value of 8A-RNase H1 at 220 nm also decreases, but only slightly, in the presence of ≥1 mM MnCl_2_, suggesting that the fraction of the N state is nearly 100% in the presence of ≥1 mM MnCl_2_.

On the assumption that the [θ] values of 6A- and 8A-RNases H1 at 220 nm in the N state are −12500 and −12000 respectively, the fraction of these proteins in the N state in the absence of MnCl_2_ is calculated to be 60% for 6A-RNase H1 and 95% for 8A-RNase H1. It is noted that the [θ] values of the mutants in the presence of 20 mM MnCl_2_ are higher than those of Halo-RNase H1 by 1000–1500. It is unlikely that this difference is caused by the difference in the protein concentration, because the protein concentration was confirmed by the method of Bradford [29] as well (data not shown). It is also unlikely that the fraction of these mutant proteins in the N state does not reach 100% even in the presence of 20 mM MnCl_2_, because the spectra of these proteins in the presence of 20 mM MnCl_2_ are nearly identical to that of Halo-RNase H1 in shape.

### Intrinsic tryptophan fluorescence spectra of Halo-RNase H1 mutants

**Figure 3:**
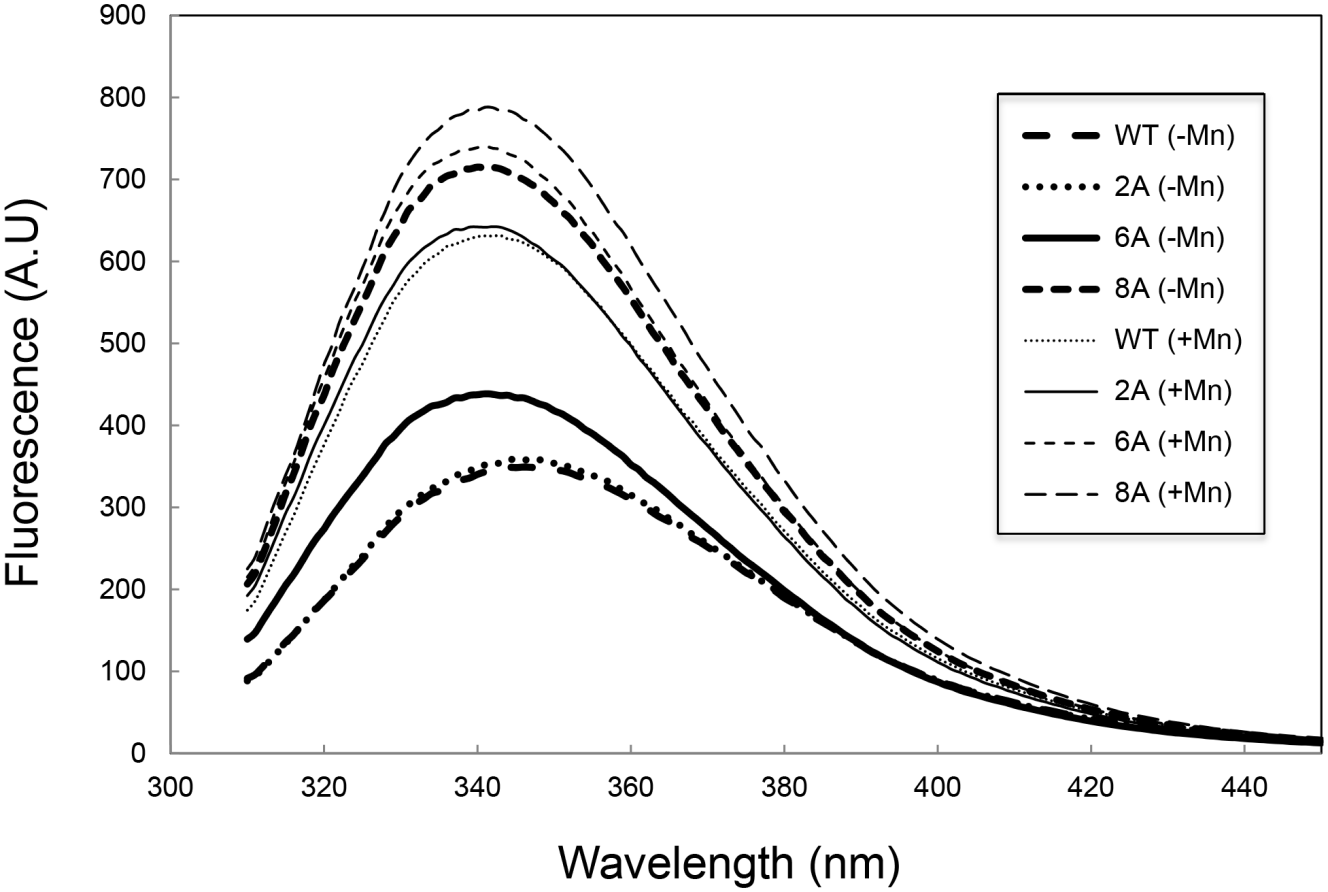
Intrinsic tryptophan fluorescence spectra of Halo-RNase H1 and its mutants. The spectra measured in the absence of salt and divalent metal ions are shown for Halo-RNase H1 (*long-dashed thick line*), 2A-RNase H1 (*dotted thick line*), 6A-RNase H1 (*solid thick line*), and 8A-RNase H1 (*short-dashed thick line*). The spectra measured in the absence of salt and presence of 10 mM MnCl_2_ are shown for Halo-RNase H1 (*long-dashed thin line*), 2A-RNase H1 (*dotted thin line*), 6A-RNase H1 (*solid thin line*), and 8A-RNase H1 (*short-dashed thin line*).

Folding of 2A-, 6A- and 8A-RNases H1 were also analyzed in the absence of salt by intrinsic tryptophan fluorescence. The intrinsic tryptophan fluorescence spectra of these proteins and Halo-RNase H1 measured in the presence of 10 mM MnCl_2_ are compared with those measured in the absence of MnCl_2_ in Figure 3. The spectra of Halo-RNase H1 and 2A-RNase H1 are similar to each other in the absence of MnCl_2_. These spectra are equally changed in the presence of 10 mM MnCl_2_, in such a way that the λ_max_ value is blue-shifted by 8 nm and the fluorescence intensity increases by approximately twice, probably due to burial of tryptophan residues inside the protein molecule. In contrast, the spectrum of 8A-RNase H1 measured in the absence of MnCl_2_ is similar to those of Halo-RNase H1 and 2A-RNase H1 measured in the presence of 10 mM MnCl_2_ and is unchanged in the presence of 10 mM MnCl_2_. The spectrum of 6A-RNase H1 measured in the absence of MnCl_2_ is different from those of other proteins in λ_max_ and/or fluorescence intensity, whereas the spectrum of 6A-RNase H1 measured in the presence of 10 mM MnCl_2_ is similar to those of other proteins. These results are consistent with those obtained by CD spectroscopy that Halo-RNase H1 and 2A-RNase H1 exist in a partially folded state (I state), and 6A- and 8A-RNases H1 partly and almost fully exist in a native state (N state) respectively in the absence of salt and MnCl_2_.

### Stability of Halo-RNase H1 mutants

We have previously shown that Halo-RNase H1 folded in a high-salt condition is stabilized in the presence of divalent metal ions [28]. *E. coli* RNase H1, which shares the similar configurations of the four acidic active site residues with Halo-RNase H1 and is folded even in a low-salt condition, is also stabilized in the presence of divalent metal ions [30]. These enzymes are probably locally destabilized due to negative charge repulsion at the active site. Crystallographic studies have indicated that one magnesium ion [31] or two manganese ions [32] bind to the active site of *E. coli* RNase H1 in a substrate-free form. Therefore, *E. coli* RNase H1 and Halo-RNase H1 are stabilized in the presence of divalent metal ions, probably because metal ion(s) binds to the active site and suppresses negative charge repulsion at this site.

2A-RNase H1 is constructed to suppress negative charge repulsion at the active site of Halo-RNase H1 and to remove the metal-binding ability of Halo-RNase H1 at the active site. However, only two of the four acidic active site residues are replaced by Ala in this mutant. To examine whether the metal-binding ability of Halo-RNase H1 at the active site is lost and negative charge repulsion at the active site is almost fully suppressed by these mutations, thermal denaturation of 2A-RNase H1 was analyzed in the presence of 2 M NaCl and in the presence and absence of 1 mM MnCl_2_ by CD spectroscopy. Thermal denaturation of Halo-RNase H1 was analyzed in those conditions for comparative purposes. Thermal denaturation of those proteins was always analyzed in a high-salt condition, because Halo-RNase H1 is folded only in a high-salt condition in the absence of divalent metal ions. Thermal denaturation of those proteins was reversible and followed a two-state mechanism in those conditions (data not shown). The *T*_m_ values of Halo-RNase H1 and 2A-RNase H1 determined in those conditions are summarized in Table 1. The *T*_m_ value of Halo-RNase H1 determined in the presence of divalent metal ions is higher than that determined in the absence of divalent metal ions by 4.5°C; whereas, the *T*_m_ value of 2A-RNase H1 determined in the presence of divalent metal ions is comparable to that determined in the absence of divalent metal ions. These results suggest that the metal-binding ability of Halo-RNase H1 at the active site is lost by the double mutations of Asp75 and Asp139 to Ala.

**Table 1:** *T*_m_ values of Halo-RNase H1 and its mutants^a^.

Protein	MnCl_2_	*T*_m_	Δ*T*_m_1^b^	Δ*T*_m_2^c^
	(mM)	(°C)	(°C)	(°C)
Halo-RNase H1	0	58.6±0.1	-	-
	1	63.1±0.1	+4.5	+4.5
2A-RNase H1	0	69.8±0.1	+11.2	-
	1	68.9±0.1	+10.3	−0.9
6A-RNase H1	0	57.2±0.2	−1.4	-
	1	62.5±1.7	+3.9	+5.3
8A-RNase H1	0	69.8±0.1	+11.2	-
	1	68.6±0.1	+10.0	−1.2

a The melting temperature (*T*_m_), which is the temperature of the midpoint of the thermal denaturation transition, was determined from the thermal denaturation curve obtained by monitoring the change in CD values at 222 nm at pH 8.0 in the presence of 2 M NaCl and either in the presence or absence of 1 mM MnCl_2_ as the temperature was increased. Errors represent those for the fitting of the curves.

b Δ*T*_m_1 is calculated as *T*_m_ of Halo-RNase H1 or its mutants determined – *T*_m_ of Halo-RNase H1 determined in the absence of MnCl_2_.

c Δ*T*_m_2 is calculated as *T*_m_ determined in the presence of 1 mM MnCl_2_ – *T*_m_ determined in the absence of MnCl_2_.

2A-RNase H1 is more stable than Halo-RNase H1 by 11.2°C in the absence of divalent metal ions (Table 1). This value is higher than but comparable to the difference between the *T*_m_ values of Halo-RNase H1 determined in the presence and absence of 20 mM MnCl_2_, which has been reported to be 9.1°C [28]. These results suggest that negative charge repulsion at the active site of Halo-RNase H1 is almost fully suppressed by the double mutations at the active site. Halo-RNase H1 is stabilized by only 4.5°C in the presence of 1 mM MnCl_2_ in a high-salt condition, probably because the active site of Halo-RNase H1 is not fully occupied by manganese ion(s) due to its relatively low binding affinity. The stability of Halo-RNase H1 and 2A-RNase H1 was not analyzed in the presence of 20 mM MnCl_2_ in this study, because thermal denaturation of 2A-RNase H1 was not reversible in this condition.

Thermal denaturation of 6A- and 8A-RNases H1 was also analyzed in the above-mentioned condition. The *T*_m_ values of these proteins determined in the presence of 1 mM MnCl_2_ and those determined in the absence of MnCl_2_ are summarized in Table 1. The stability of 6A-RNase H1 is similar to that of Halo-RNase H1 either in the presence or absence of MnCl_2_. Both proteins are equally stabilized in the presence of MnCl_2_. Likewise, the stability of 8A-RNase H1 is similar to that of 2A-RNase H1 either in the presence or absence of MnCl_2_. Both proteins are equally more stable than Halo-RNase H1 in the absence of MnCl_2_ but are not further stabilized in the presence of MnCl_2_. These results indicate that sextuple mutations at the bi/quad-aspartate sites do not significantly affect the protein stability in a high-salt condition and therefore binding of divalent metal ion(s) to these sites cannot be analyzed by monitoring the change in protein stability in this condition. Therefore, it remains to be determined whether metal ions bind to the bi/quad-aspartate sites of Halo-RNase H1 and suppress negative charge repulsion at these sites that prevent folding of Halo-RNase H1 in a low-salt condition. To identify the binding sites of divalent metal ions that are responsible for folding of Halo-RNase H1 in a low-salt condition, it will be necessary to determine the crystal structure of 2A-RNase H1 folded in the presence of divalent metal ions. 2A-RNase H1 does not have the active site, to which divalent metal ion(s) binds, but still requires divalent metal ions for folding.

### Enzymatic activity of 6A-RNase H1

**Figure 4:**
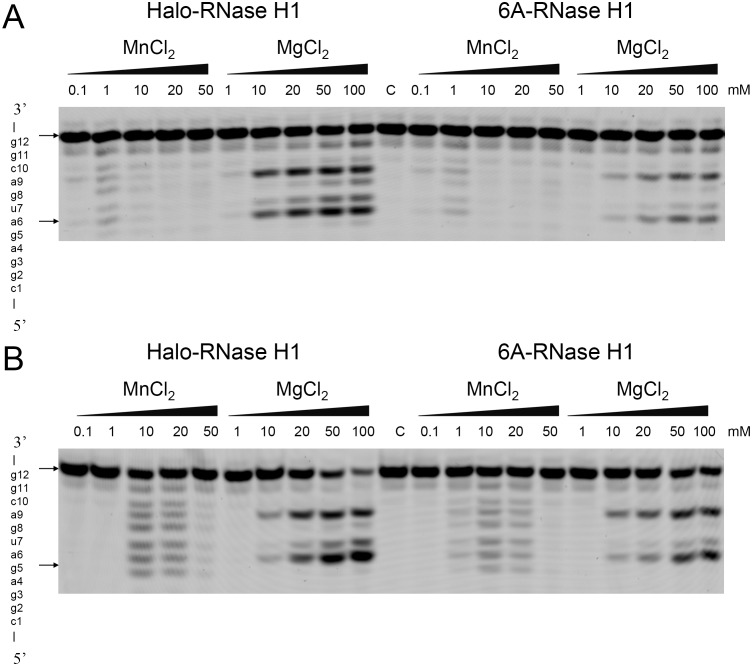
Cleavage of R12/D12 substrate with Halo-RNase H1 and 6A-RNase H1. The 5′-end labeled R12/D12 substrate was hydrolyzed by Halo-RNase H1 or 6A-RNase H1 at 37°C for 15 min in the presence of various concentrations of MnCl_2_ or MgCl_2_ and in the presence of 50 mM (B) or 3 M (A) NaCl. The hydrolysates were separated on a 20% polyacrylamide gel containing 7 M urea. The concentration of the substrate was 1 µM. The amount of the enzyme added to the reaction mixture (10 µl) was 1 ng. The enzyme and divalent metal ions used to hydrolyze the substrate are shown above the gel together with the concentrations of the divalent metal ions. The sequence of R12 is indicated along the gel.

**Figure 5:**
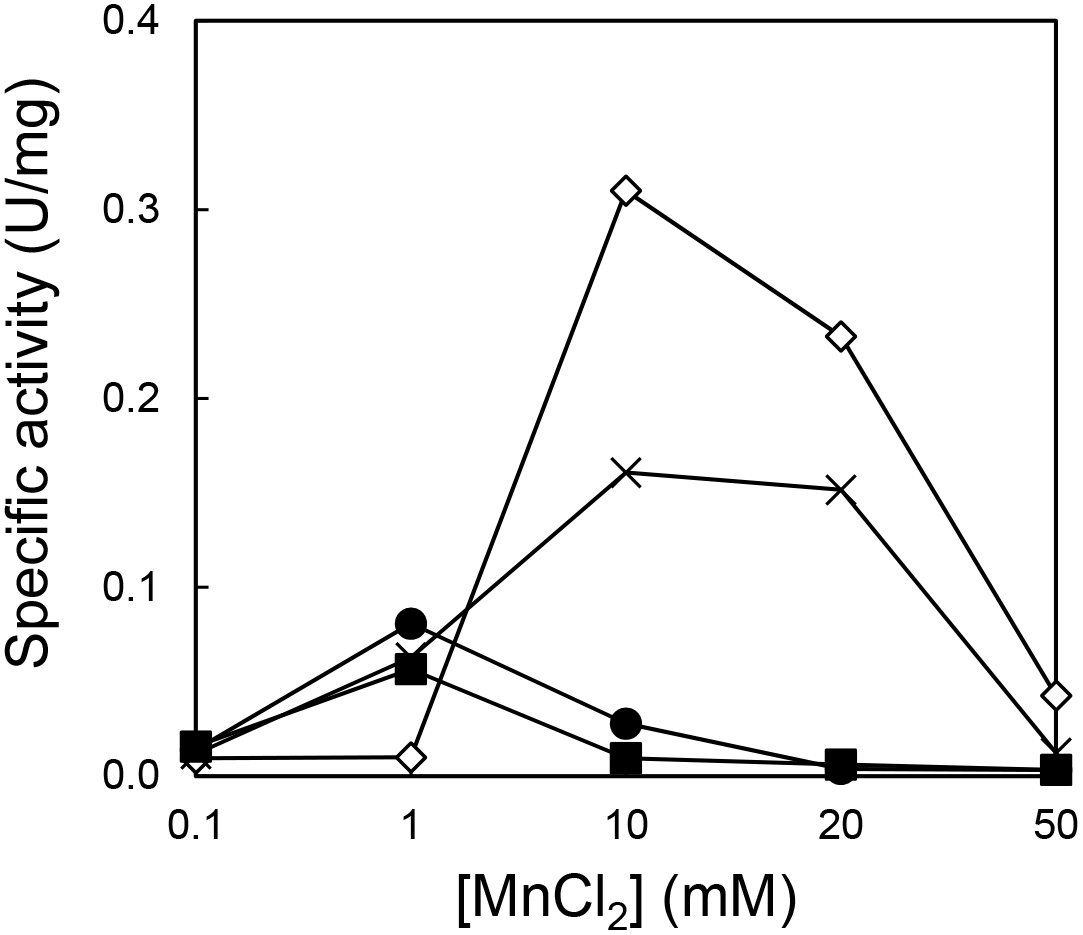
Manganese dependencies of Halo-RNase H1 and 6A-RNase H1 activities. The specific activities of Halo-RNase H1 in the presence of 50 mM (open diamond) and 3 M (closed circle) NaCl and those of 6A-RNase H1 in the presence of 50 mM (cross) and 3 M (closed square) NaCl, which are estimated from the gels shown in Figure 3, are plotted as a function of the MnCl_2_ concentration.

To examine whether the mutations at the bi/quad-aspartate sites affect the enzymatic activity of Halo-RNase H1, the activities of Halo-RNase H1 and 6A-RNase H1 were determined in the presence of 50 mM or 3 M NaCl using the R12/D12 substrate. The concentration of MnCl_2_ or MgCl_2_ varied from 0.1 to 50 or 1 to 100 mM respectively. The separation of oligoribonucleotides produced upon hydrolysis of the substrate in the presence of 50 mM or 3 M NaCl on a urea gel is shown in Figure 4. Both Halo-RNase H1 and 6A-RNase H1 similarly cleaved the substrate at all sites between a4 and g11 in the presence of manganese ions and at four sites between a6 and c10, preferably at a6-u7 and a9-c10, in the presence of magnesium ions. The specific activities of these proteins in the presence of various concentrations of MnCl_2_ were estimated by comparing the intensity of the band of the substrate shown in Figure 4, which remains uncleaved, and were plotted as a function of MnCl_2_ concentration in Figure 5. Both proteins exhibit the highest activities in the presence of 50 mM NaCl and 10 mM MnCl_2_. In the presence of 3 M NaCl, both proteins exhibit the highest activities in the presence of 1 mM MnCl_2_. The specific activity of 6A-RNase H1 is lower than that of Halo-RNase H1 by 50% in the presence of 50 mM NaCl and 10 mM MnCl_2_ and 30% in the presence of 3 M NaCl and 1 mM MnCl_2_. This result suggests that the sextuple mutations at the bi/quad-aspartate sites reduce the activity of Halo-RNase H1 by 30–50%.

Halo-RNase H1 exhibits little activity in the presence of 1 mM MnCl_2_ in a low-salt condition (50 mM NaCl), because the fraction of the protein in the N state is low (∼20%) in this condition [28]. In contrast, 6A-RNase H1 exhibits comparable activity to that determined in the presence of 3 M NaCl and 1 mM MnCl_2_, even in the presence of 50 mM NaCl and 1 mM MnCl_2_, probably because the fraction of this protein in the N state increases to nearly 90% in this condition (Fig. 2B).

The activities of Halo-RNase H1 and 6A-RNase H1 determined in the presence of 3 M NaCl and 1 mM MnCl_2_ are approximately 30% of those determined in the presence of 50 mM NaCl and 10 mM MnCl_2_. These activities greatly decrease when the MnCl_2_ concentration increases to 10 mM (Fig. 5). In contrast, the activities of Halo-RNase H1 and 6A-RNase H1 determined in the presence of 3 M NaCl and 10 mM MgCl_2_ do not significantly change or rather increase when the MgCl_2_ concentration increases to 100 mM. This discrepancy is probably caused by the difference in the binding affinities of the manganese and magnesium ions to the active site of Halo-RNase H1. Optimum concentration of the manganese ions for the activity of Halo-RNase H1 is apparently significantly lower than that of the magnesium ions (Fig. 4), suggesting that the binding affinity of the manganese ion to the active site of Halo-RNase H1 is higher than that of the magnesium ions. If the MnCl_2_ concentration exceeds the optimum one for activity, additional manganese ion(s) may bind to the active site and may inhibit the activity of Halo-RNase H1.

### Amino acid sequence of Halo-RNase H2

**Figure 6:**
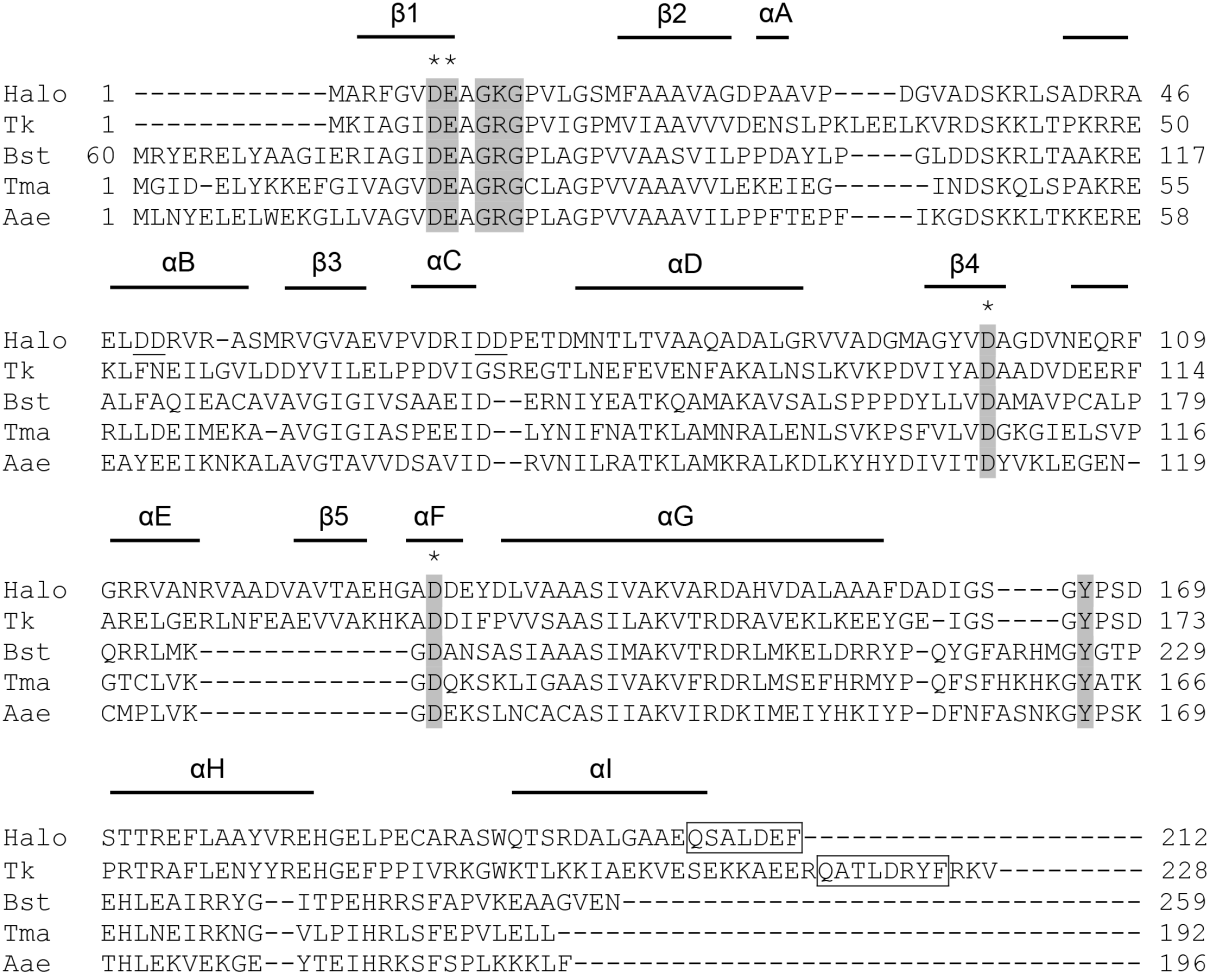
Alignment of the amino acid sequences. The amino acid sequences of Halo-RNase H2 (Halo), Tk-RNase H2 (Tk), Bst-RNase H2 (Bst), Tma-RNase H2 (Tma), and Aae-RNase H2 (Aae) are compared with one another. The accession numbers are AE004437 for Halo-RNase H2, AB012613 for Tk-RNase H2, BAB91155 for Bst-RNase H2, AAD35996 for Tma-RNase H2, and AAC07736 for Aae-RNase H2. The ranges of the secondary structures of Halo-RNase H2 deduced from its tertiary model are shown above the sequences. The four acidic active site residues are shaded and marked with asterisks. A GRG or GKG motif and a tyrosine residue that are responsible for recognition of a single ribonucleotide misincorporated into dsDNA are shaded. The dashes stand for the gaps. The numbers represent the positions of the amino acid residues relative to the initiator methionine for each protein. PCNA interacting peptide (PIP) motif is boxed.

Halo-RNase H2 is composed of 212 amino acid residues with a calculated molecular mass of 22,109 Da and an isoelectric point (p*I*) of 4.6. The amino acid sequence of Halo-RNase H2 is compared with those of the representative members of prokaryotic RNases H2, such as RNases H2 from *Thermococcus kodakarensis* (Tk-RNase H2), *Thermotoga maritima* (Tma-RNase H2), *Bacillus stearothermophilus* (Bst-RNase H2), and *Aquifex aeolicus* (Aae-RNase H2) in Figure 6. Halo-RNase H2 shows the amino acid sequence identities of 39% to Tk-RNase H2, 22% to Tma-RNase H2, 25% to Bst-RNase H2 (without N-terminal extension), and 25% to Aae-RNase H2. Unlike Halo-RNase H1 and Bst-RNase H2, Halo-RNase H2 possesses neither N- nor C-terminal extension. Four acidic active site residues that form the metal binding site are conserved as Asp7, Glu8, Asp100, and Asp130 in Halo-RNase H2. A GRG motif and a tyrosine residue that are responsible for recognition of a single ribonucleotide misincorporated into double-stranded DNA (dsDNA) [26] are not fully conserved but are conserved as a GKG motif (Gly10, Lys11, and Gly12) and Tyr166 in Halo-RNase H2. This GKG motif is conserved in *Archaeoglobus fulgidus* RNase H2 and *Chlamydophila pneumoniae* RNase H3, both of which exhibit an ability to cleave dsDNA containing a single ribonucleotide [33]. Furthermore, Halo-RNase H2 contains a proliferating cell nuclear antigen (PCNA) interacting peptide (PIP) box motif at the C-terminus, suggesting that Halo-RNase H2 interacts with PCNA at this region.

### Tertiary Model of Halo-RNase H2

**Figure 7:**
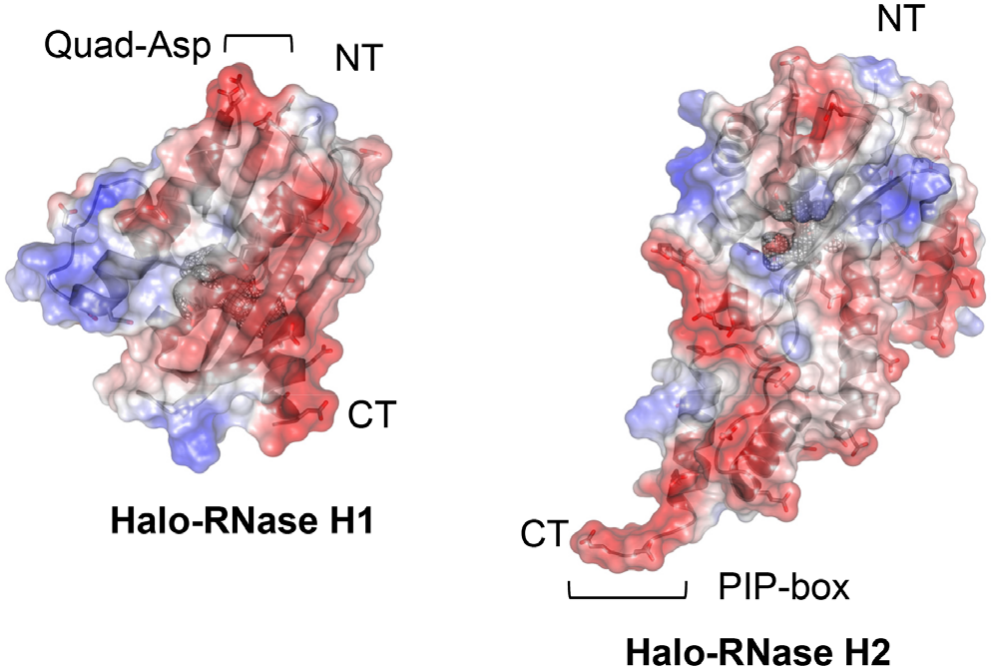
A tertiary model of Halo-RNase H2. Ribbon diagram with electrostatic surface potential of Halo-RNase H2 is shown in comparison with that of Halo-RNase H1. NT and CT represent N and C-termini. The negative and positive potentials are in red and blue respectively. The electrostatic potential value ranges from −100 to +100 kT e^−1^. The positions of quad-aspartate site (Quad-Asp) of Halo-RNase H1 and PIP-box of Halo-RNase H2 are shown.

Halo-RNase H2 is characterized by the high content (18.4%) of acidic amino acid residues. A tertiary model of this protein shows the abundance of acidic residues on the surface as does the crystal structure of Halo-RNase H1 (Fig. 7). These acidic residues include those of the two bi-aspartate sites (Asp49-Asp50 and Asp69-Asp70). As a result, the surface of Halo-RNase H2 is negatively charged, as is that of Halo-RNase H1. The overall structure of Halo-RNase H2 is similar to that of Tk-RNase H2 (PDB code 2dfh). The steric configurations of the four acidic active site residues are similar to those of Tk-RNase H2 (data not shown).

### Preparation of Halo-RNase H2

To examine whether Halo-RNase H2 also requires salt or divalent metal ions for folding, Halo-RNase H2 was overproduced in *E. coli* as a glutathione S-transferase (GST)-Halo-RNase H2 fusion protein. Upon overproduction, this fusion protein accumulated in the cells in a soluble form. Halo-RNase H2 was released from the fusion protein by on-column cleavage with thrombin and was purified to give a single band on SDS-PAGE (data not shown). The amount of the protein purified from 1 L culture was typically 2 mg. Attempts to overproduce Halo-RNase H2 alone have so far been unsuccessful, because the production level of this protein is too low to purify it in an amount sufficient for biochemical characterization.

### Folding of Halo-RNase H2

**Figure 8:**
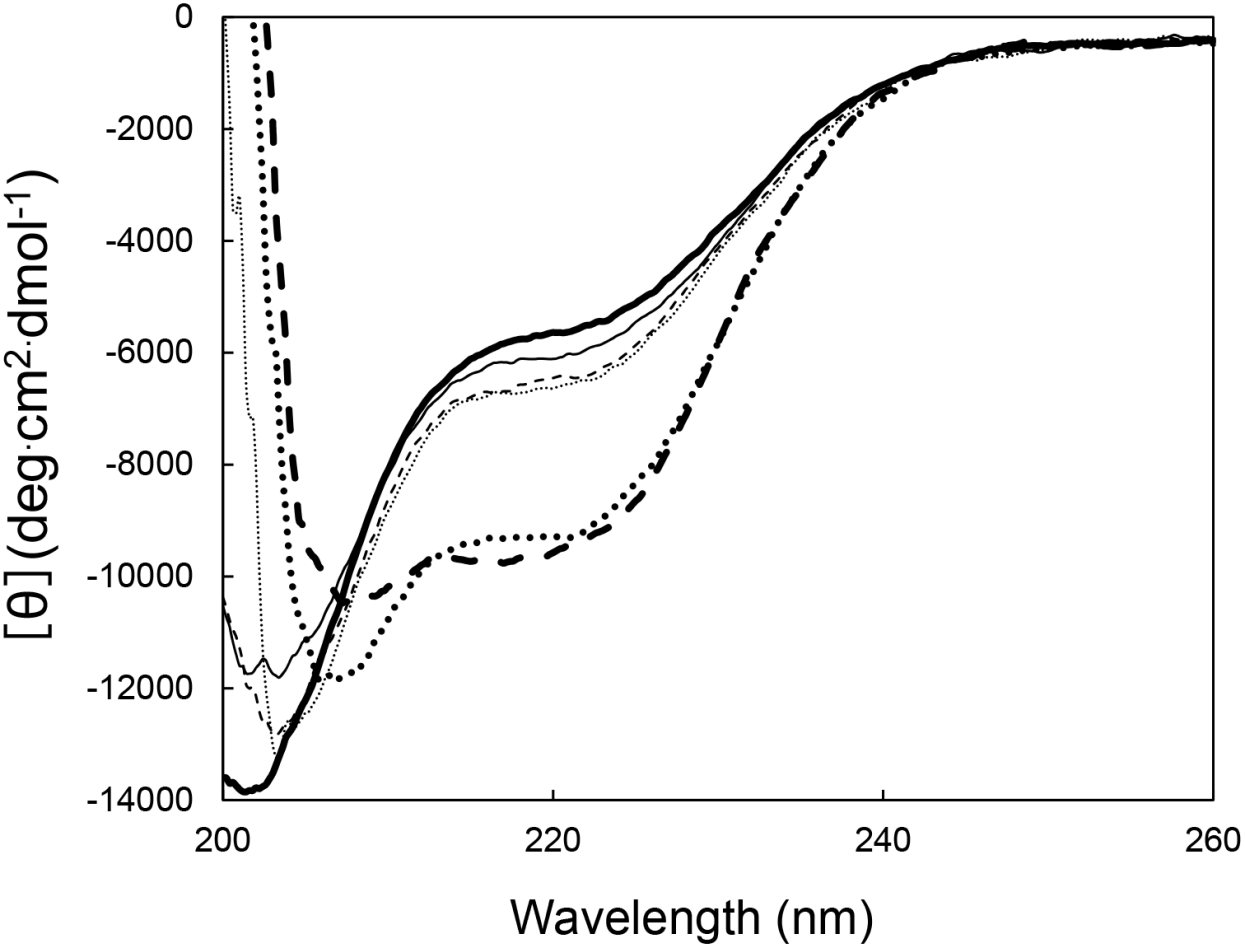
CD spectra of Halo-RNase H2. The far-UV CD spectra of Halo-RNase H2 measured at 25°C and pH 8.0 in the absence of salt and divalent metal ions (*solid thick line*), in the absence of salt and the presence of 10 mM MnCl_2_ (*solid thin line*), 20 mM MnCl_2_ (*dashed thin line*), and 500 mM MgCl_2_ (*dotted thin line*), and in the presence 3 M NaCl (*dotted thick line*) and 4 M NaCl (*dashed thick line*) and the absence of divalent metal ions are shown.

Folding of Halo-RNase H2 was analyzed by CD spectroscopy. The far-UV CD spectra of Halo-RNase H2 measured at 25°C and pH 8.0 in the absence or presence of various concentrations of NaCl or divalent metal ions are shown in Figure 8. In the absence of salt and divalent metal ions, the far-UV CD spectrum of Halo-RNH2 gives a trough with a minimum [θ] value of −14,000 at 202 nm, which is accompanied by a shoulder with a [θ] value of −6000 at 220 nm. This spectrum is greatly changed in the presence of 3 M NaCl, in such a way that it gives a trough with a minimum [θ] value of −12000 at 208 nm and the [θ] value at 220 nm decreases to −9500. This spectrum is changed when the NaCl concentration increases from 3 to 4 M, but only slightly, suggesting that Halo-RNase H2 is almost fully folded in the presence of 3 M NaCl. In contrast, the spectrum of Halo-RNase H2 measured in the absence of salt and divalent metal ions does not significantly change even in the presence of 20 mM MnCl_2_ or 500 mM MgCl_2_. These results suggest that Halo-RNase H2 is folded in a high-salt condition, but is not folded in a low-salt condition regardless of the presence of divalent metal ions. Thus, we conclude that requirement of divalent metal ions for protein folding as a substitute of salt is unique to Halo-RNase H1.

### Enzymatic activity of Halo-RNase H2

**Figure 9:**
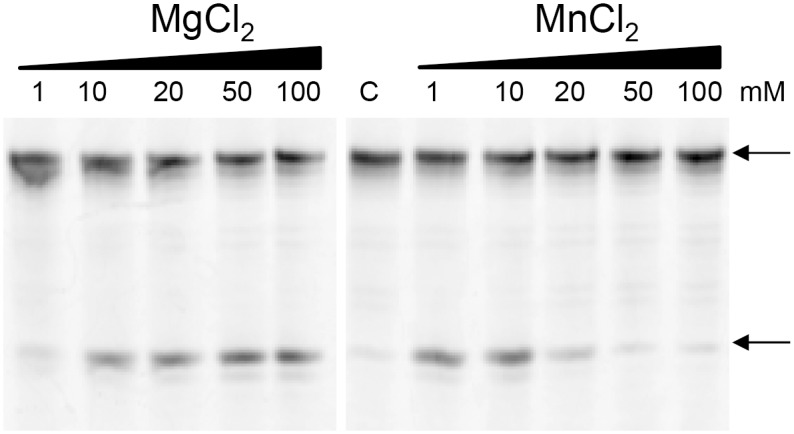
Cleavage of DNA15-RNA1-DNA13/DNA29 substrate with Halo-RNase H2. The 5′-end labeled DNA15-RNA1-DNA13/DNA29 substrate was hydrolyzed by Halo-RNase H2 at 37°C for 15 min in the presence of 3 M NaCl and various concentrations of MnCl_2_ or MgCl_2._ The hydrolysates were separated on a 20% polyacrylamide gel containing 7 M urea. The concentration of the substrate was 1 µM. The amount of the enzyme added to the reaction mixture (10 µl) was 500 ng. Divalent metal ions used to hydrolyze the substrate and the concentrations of these divalent metal ions are shown above the gel. The arrows indicate the 5′-end labeled substrate (top) and 5′-end labeled 15-mer DNA (bottom).

To examine whether Halo-RNase H2 exhibits JRNase activity, the enzymatic activity of Halo-RNase H2 was determined in the presence of 50 mM or 3 M NaCl and in the presence of various concentrations of MnCl_2_ or MgCl_2_ at 37°C using the 5′-fluorescein-labeled D15-R1-D13/D29 substrate. The concentration of MnCl_2_ or MgCl_2_ varied from 1 to 100 mM. The separation of the oligonucleotide (5′-fluorescein-labeled 15-mer DNA) produced upon hydrolysis of the substrate in the presence of 3 M NaCl on a urea gel is shown in Figure 9. Halo-RNase H2 was able to cleave the substrate at the (5′)DNA-RNA(3′) junction in the presence of 3 M NaCl and 10–100 mM MgCl_2_ or 1–10 mM MnCl_2_. Halo-RNase H2 was unable to cleave the substrate in a low-salt condition at any concentration of MnCl_2_ or MgCl_2_ that was examined (data not shown). These results are consistent with those obtained by CD spectroscopy that Halo-RNase H2 is partially folded in a low-salt-condition even in the presence of 20 mM MnCl_2_ or 500 mM MgCl_2_, but is almost fully folded in the presence of 3 M NaCl.

### Divalent metal ion-induced folding mechanism of Halo-RNase H1

Comparison of the crystal structure of Halo-RNase H1 and a tertiary model of Halo-RNase H2 indicates that both proteins are characterized by the high content of acidic residues on the surface and share the similar configurations of the four acidic active site residues. The configurations of these active site residues are conserved in all RNases H so far examined [5]. The findings that one or two divalent metal ions bind to the active sites of *E. coli* RNase H1 [31], [32], [34], HIV-1 RNase H [35], and *B. stearothermophilus* RNase H3 [36] in a substrate-free form and two divalent metal ions bind to the active sites of *B. halodurans* RNase H1 [37], human RNase H1 [38], and *T. maritima* RNase H2 [27] in a substrate-bound form suggest that at least one divalent metal ion binds to the active sites of Halo-RNase H1 and Halo-RNase H2 in a substrate-free form when they are folded. However, Halo-RNase H2 is not folded in the presence of divalent metal ions in a low-salt condition. As mentioned above, 2A-RNase H1, in which negative charge repulsion at the active site is almost fully suppressed by the mutations, is also not folded in a low-salt condition. These results suggest that suppression of negative charge repulsion at the active site by binding of divalent metal ion(s) is not sufficient to induce folding of Halo-RNase H1 and Halo-RNase H2. When the localization of the aspartate residues on the surface of Halo-RNase H1 is compared with that of Halo-RNase H2, a quad-aspartate site is only present on the surface of Halo-RNase H1.

The structure of the quad-aspartate site of Halo-RNase H1 is shown in Figure 1B. According to the crystal structure of Halo-RNase H1 folded in the presence of 10 mM MnCl_2_, two molecules (molecules A and B) contact each other within the crystal lattice. Molecule A of one crystal lattice also contacts molecules A′ and A″ of other crystal lattices. Two quad-aspartate site residues (Asp173 and Asp174) of molecule A provide ligands for coordination of two manganese ions (Mn1 and Mn2), which are also coordinated with the four acidic active site residues of molecule A″. In contrast, any quad-aspartate residue of molecule B does not provide any ligand for coordination of manganese ions. Comparison of the structures of the quad-aspartate sites, to which manganese ions bind and do not bind, indicates that the side chains of the quad-aspartate site residues, especially Asp173, greatly change their positions upon binding of manganese ions (Fig. 1B). This result suggests that these residues are highly flexible and greatly change the positions of their side chains, in such a way that a manganese ion binds to the middle of this site in solution.

We propose that suppression of negative charge repulsion at the quad-aspartate site by binding of divalent metal ion is required to initiate folding of Halo-RNase H1 and that at the active site is required to stabilize the folded state of this protein. The results that 6A- and 8A-RNases H1 partly and almost fully exist in the N state respectively and the concentration of MnCl_2_ required to induce folding of 2A-RNase H1 is significantly lower than that required to induce folding of Halo-RNase H1 support this proposal. As far as we know, none of other halophilic proteins so far examined is folded in a metal-dependent manner [39]–[48]. The physiological significance of metal-dependent folding of Halo-RNase H1 remains to be understood, because other proteins from the same organism, including Halo-RNase H2, are not folded in a metal-dependent manner [42], [43].

## Materials and Methods

### Plasmid construction

The pET25b derivatives for overproduction of the Halo-RNase H1 mutants, 2A, 6A-, and 8A-RNases H1, were constructed by PCR using the KOD-Plus Mutagenesis kit (Toyobo, Kyoto, Japan) according to the manufacturer’s instructions. The pET25b derivative for overproduction of Halo-RNase H1, which was previously constructed [28], was used as a template. The mutagenic primers were designed in such a way that the GAC and GAT codons for Asp are changed to GCC and GCT for Ala respectively, except for the GAC codon for Asp197, which is changed to the GCA codon for Ala.

Plasmid pGEX-Halo-RNH2 for overproduction of the glutathione S-transferase (GST)-Halo-RNase H2 fusion protein, in which Halo-RNase H2 is attached to the C-terminus of GST through a linker containing the thrombin cleavage site, was constructed by ligating the DNA fragment amplified by PCR into the *Bam*HI-*Eco*RI sites of the pGEX-2T vector (GE Healthcare, Little Chalfont, Buckinghamshire, England). The genomic DNA of *Halobacterium* sp. NRC-1, which was kindly donated by Dr. M. Tokunaga, was used as a template. The sequences of the PCR primers are 5′-CGCGGATCCATGGCGCGGTTCGG-3′ for the 5′-primer and 5′-CCGGAATTCCGGTTATCAAAACTCGTCCAG-3′ for the 3′-primer, where the *Bam*HI (5′-primer) and *Eco*RI (3′-primer) sites are underlined.

All DNA oligomers for PCR were synthesized by Hokkaido System Science (Sapporo, Japan). PCR was performed with a GeneAmp PCR system 2400 (Applied Biosystems, Tokyo, Japan). The DNA sequences were confirmed by a Prism 310 DNA sequencer (Applied Biosystems).

### Overproduction and purification

*E. coli* BL21-CodonPlus(DE3) (Stratagene, La Jolla, CA, USA) was used as a host strain for overproduction of the Halo-RNase H1 mutants and GST-Halo-RNase H2 fusion protein. The transformants of this strain with the pET25b derivatives and pGEX-Halo-RNH2 were grown at 37°C in LB medium containing 50 µg^.^mL^−1^ ampicillin and 30 µg^.^mL^−1^ chloramphenicol. When the absorbance at 600 nm reached approximately 0.5, 1 mM isopropyl thio-β-D-galactoside (IPTG) was added to the culture medium and cultivation was continued at 37°C for an additional 4 h. The subsequent purification procedures of the Halo-RNase H1 mutants and Halo-RNase H2 were carried out at 4°C.

Halo-RNase H1 and its mutants (2A-, 6A-, and 8A-RNases H1) were purified as previously described for Halo-RNase H1 [28], except that the buffer for the Mono Q column step is changed from 20 mM sodium acetate (pH 5.5) to 10 mM Tris-HCl (pH 7.0).

For purification of Halo-RNase H2, cells were harvested by centrifugation at 8000 *g* for 10 min, suspended in 10 mM sodium phosphate (pH 7.3) containing 140 mM NaCl, 2.7 mM KCl, and 2 mM DTT (buffer A), lysed by sonication, and centrifuged at 30,000 *g* for 30 min. The supernatant was collected, dialyzed against buffer A, and loaded onto a GSTrap HP column (5 ml) (GE Healthcare) equilibrated with the same buffer. On-column thrombin cleavage of the GST-Halo-RNase H2 fusion protein was performed by loading thrombin dissolved in 5 mL of buffer A onto the column and incubating the column at 15°C for 20 min. The amount of thrombin loaded onto the column was 8% (w/w) of that of the fusion protein loaded onto the column. The Halo-RNase H2 molecules released from the fusion protein upon thrombin cleavage were eluted from the column by buffer A. GST was afterward eluted by 50 mM Tris-HCl (pH 8.0) containing 10 mM reduced glutathione (Wako Pure Chemical Industries, Ltd., Osaka, Japan). The fractions containing Halo-RNase H2 were collected, dialyzed against 10 mM Tris-HCl (pH 7.0) containing 1 mM EDTA, and applied to a Mono Q column (1 ml) (GE Healthcare) equilibrated with the same buffer. The protein was eluted from the column with a linear gradient of NaCl from 0 to 1 M. The fractions containing the protein were collected and loaded to a HiLoad 16/600 Superdex 200 pg column (GE Healthcare) equilibrated with 10 mM Tris-HCl (pH 8.0). The fractions containing the protein were collected and used for biochemical characterization.

The purity of the protein was analyzed by SDS-PAGE [49] using a 15% (w/v) polyacrylamide gel, followed by staining with Coomassie Brilliant Blue (CBB). The protein concentration was determined from UV absorption using a cell with an optical path length of 1 cm and an *A*_280_ value for 0.1% (1.0 mg^.^mL^−1^) solution of 1.26 for Halo-RNase H1 and its derivatives and 0.49 for Halo-RNase H2. These values were calculated by using absorption coefficients of 1576 M^−1.^cm^−1^ for Tyr and 5225 M^−1.^cm^−1^ for Trp at 280 nm [50].

### CD spectra

The far-UV CD spectra (200–260 nm) were measured on a J-725 spectropolarimeter (Japan Spectroscopic Co., Ltd., Tokyo, Japan) at 25°C. The protein was dissolved in 10 mM Tris-HCl (pH 8.0) containing various concentrations of MnCl_2_. The protein concentration was approximately 0.1 mg^.^mL^−1^ and a cell with an optical path length of 2 mm was used. The mean residue ellipticity, θ, which has the units of deg^.^cm^−2.^dmol^−1^, was calculated by using an average amino acid molecular mass of 110 Da.

### Tryptophan fluorescence

The intrinsic tryptophan fluorescence was measured with a Shimadzu RF-5300PC Spectrofluorophotometer (Shimadzu, Kyoto, Japan). The excitation wavelength was 295 nm, the emission wavelength scan was done at 310–450 nm with a medium scan rate. The slit widths of the excitation and emission monochromators were set at 5.0 and 3.0 nm respectively. The spectra were measured at 25°C and pH 8.0 in the absence or presence of 10 mM MnCl_2_. The protein concentration was 0.1 mg^.^mL^−1^.

### Thermal denaturation

Thermal denaturation of the protein was analyzed by monitoring the change in CD values at 222 nm as the temperature was increased. The protein was dissolved in 10 mM Tris-HCl (pH 8.0) containing 2 M NaCl or the same buffer containing 2 M NaCl and 1 mM MnCl_2_. The protein concentration and optical path length were 0.1 mg^.^mL^−1^ and 2 mm respectively. The temperature of the protein solution was linearly increased by approximately 1.0°C^.^min^−1^. Thermal denaturation of the protein was reversible at the conditions examined. The temperature of the midpoint of the transition, *T*_m_, was calculated from curve fitting of the resultant CD values versus temperature data on the basis of a least squares analysis.

### Enzymatic activity

The RNase H and JRNase activities were determined by using 5′-fluorescein-labeled RNA/DNA hybrid (R12/D12) and 29 bp DNA15-RNA1-DNA13/DNA29 (D15-R1-D13/D29) as substrates respectively, as described previously [51], except that the hydrolytic reaction was performed at 37°C instead of 30°C. The reaction buffer was 10 mM Tris-HCl (pH 8.5) containing 1 mM DTT, 0.01% BSA, 50 mM or 3 M NaCl, and various concentrations of MgCl_2_ or MnCl_2_. The substrate concentration was 1 µM. The products were detected by Typhoon 9240 Imager (GE Healthcare) and quantified using Image Quant 5.2 analysis software. One unit is defined as the amount of enzyme degrading 1 µmol of the substrate per min at 37°C. The specific activity was defined as the enzymatic activity per milligram of protein.

### Homology modeling

A model for the three-dimensional structure of Halo-RNase H2 was built by SWISS-MODEL – an automated protein homology-modeling server - (Swiss Institute of Bioinfomatics) [52], using the structure of Tk-RNase H2 (PDB ID: 2dfh) as a template. These proteins share the amino acid sequence identity of 38.8%. The model was viewed and edited with PyMOL (www.pymol.org).
